# Concordance of the spectral properties of dorsal wing scales with the phylogeographic structure of European male *Polyommatus icarus* butterflies

**DOI:** 10.1038/s41598-021-95881-z

**Published:** 2021-08-13

**Authors:** Gábor Piszter, Krisztián Kertész, Gábor Sramkó, Virág Krízsik, Zsolt Bálint, László Péter Biró

**Affiliations:** 1 Institute of Technical Physics and Materials Science, Centre for Energy Research, P.O. Box 49, 1525 Budapest, Hungary; 2 MTA-DE “Lendület” Evolutionary Phylogenomics Research Group, 1 Egyetem Sq., 4032 Debrecen, Hungary; 3 Department of Zoology, Hungarian Natural History Museum, 13 Baross St., 1088 Budapest, Hungary

**Keywords:** Photonic crystals, Photonic crystals, Evolutionary biology

## Abstract

The males of more than 80% of the Lycaenidae species belonging to the tribe Polyommatini exhibit structural coloration on their dorsal wing surfaces. These colors have a role in reinforcement in prezygotic reproductive isolation. The species-specific colors are produced by the cellular self-assembly of chitin/air nanocomposites. The spectral position of the reflectance maximum of such photonic nanoarchitectures depends on the nanoscale geometric dimensions of the elements building up the nanostructure. Previous work showed that the coloration of male *Polyommatus icarus* butterflies in the Western and Eastern Palearctic exhibits a characteristic spectral difference (20 nm). We investigated the coloration and the de novo developed DNA microsatellites of 80 *P. icarus* specimens from Europe from four sampling locations, spanning a distance of 1621 km. Remarkably good concordance was found between the spectral properties of the blue sexual signaling color (coincident within 5 nm) and the population genetic structure as revealed by 10 microsatellites for the *P. icarus* species.

## Introduction

The wings of butterflies exhibit a richness of colors and patterns unrivalled in the living world^1–4^. Many of these colors are efficiently used and optimized in sexual communication. These colors may be generated by selective light absorption on pigments, by selective light reflection on photonic nanoarchitectures, or by a combination of the two^5,6^. Structural colors started to evolve 200 million years ago^7^ and fossilized moth scales of 47 million years already exhibit structures resembling the scales of extant lepidopterans^8^. The structural colors are generated by the interaction of light with nanoarchitectures with typical dimensions in the wavelength range of the visible light^9^. Surprisingly, even the apparently colorless insect wings may generate structural color, so called “wing interference patterns”, for example in the transparent wings of small Hymenoptera and Diptera, patterns that have largely been overlooked by biologists^10^. Structural coloration is a striking component of sexual ornamentation and may function as a signal of mate quality^11^.

The males of many Gossamer-winged butterflies (Lepidoptera: Papilionoidea: Lycaenidae)—one of the most speciose butterfly families with 416 genera and 5201 species^12^—possess species-specific blue sexual signaling colors of structural origin^13,14^. The importance of male dorsal wing coloration in natural selection has been stressed by the role of coloration in prezygotic isolation mechanisms, such as reinforcement^15^. In a study on *Polyommatus* Latreille, 1804 subgenus *Agrodiaetus* Hübner, 1822 from 140 species belonging to the tribe Polyommatini (Polyommatinae), only 26 (18.5%) species were found with brown dorsal coloration of the males, all the other exhibiting structural coloration^16^.

Lepidopteran structural colors arise from species-specific, chitin based photonic nanoarchitectures of the dorsal cover scales^9,17–20^. Although some intraspecific variation may be present in the optical properties of the nanoarchitectures—as in any biological structure—the natural variation in the spectral position of the reflectance maximum for a given population locally is surprisingly small, for example, in the species *Polyommatus icarus* (Rottemburg, 1775), one of the most common blue butterflies of Europe^21^, it is of the order of only 10 nm^22^. Both the reflectance spectra and the geometric parameters of the photonic nanoarchitectures allow species identification with an accuracy of 96% and 91%, respectively^13,14^.

**Figure 1:**
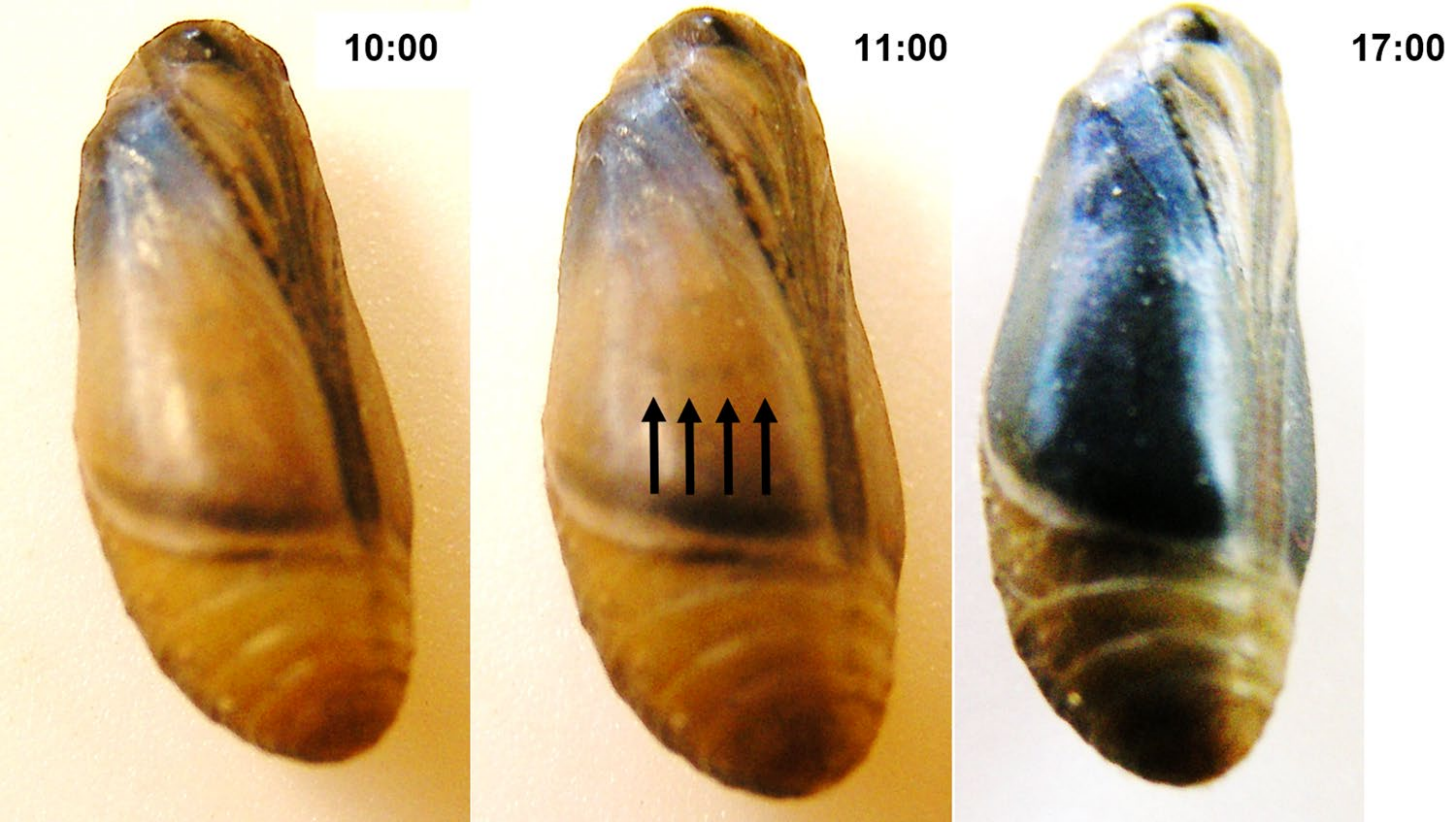
Pupa of a *Polyommatus icarus* male specimen one day prior to the eclosion of the adult. In the morning hours, the dorsal wing surface visible through the pupal case has a light brown-yellow color, through which the dark spot array from the ventral wing side is visible (arrows in the central image). This indicates that the scales of the dorsal wing surface are still transparent. In the afternoon, the color change is completed, the entire dorsal wing surface appears blue.

Lepidopteran wing scales and the intricate color generating photonic nanoarchitectures are produced by specialized cells^23–27^. The photonic nanoarchitectures are self-assembled from chitin during metamorphosis inside the living scale cell. Early stages of scale growth are observed as early as five hours after pupation^28^, while the final stage of scale formation can be observed for example for male *Polyommatus icarus*, regularly on the day prior to eclosion. This is indicated by the turning of the color seen inside the pupa of the males from light brown to the structural blue, see Fig. 1.

The remarkable structural and spectral reproducibility in the macroscopic realm of the species-specific properties of the photonic nanoarchitectures inside the dorsal cover scales is achieved via self-assembly occurring at microscopic level, individually in each cover scale. The exact mechanism is still debated^23,25,26,29,30^. The processes taking place inside the cells are governed by the DNA located in the cell nucleus. Although the nanometer scale processes are difficult to visualize in living cells, the micron scale processes revealed by confocal and multiphoton microscopy with fluorescent dye staining indicate that the actin filaments have a decisive role in shaping the micron scale system of chitin ridges of the butterfly scales, defining the architecture in this size range^25^. Inside cells, actin and various actin bundling proteins are responsible for building of the cytoskeleton, filopodia, microvilli, etc.^31–33^. Very likely, the mold in which the chitin of the species-specific photonic nanoarchitecture is shaped is also built from these proteins.

Recently, we investigated the biogeographic patterns in the structural blue coloration of *Polyommatus icarus* males in Europe and Asia^34^. In agreement with earlier allozyme based investigations, it was found that the males originating from the western part of Europe were not divided into distinct lineages^35^. Variations of the ventral wing patterns also delineated a western and an eastern group (based on 16 marker characters)^36^. However, investigations based on the mitochondrial barcoding region, a 650 base pair (bp) fragment of the mitochondrial cytochrome-oxidase I (COI) gene at its 3’ end, revealed five main European lineages: a large Palearctic lineage that prevailed the distribution area, with Iberia and Italy included; Sierra Nevada and Alicante-Provence^37,38^. Although COI could not separate lineages within the Palearctic lineage (as defined by Dincă et al.^37^), clear differences in coloration were found between the European and Asian group of samples, with a transitional zone in the Anatolian region of Turkey^34^. The results of mitochondrial and nuclear DNA may show discordance^39,40^ and their phylogenetic resolution can also be markedly different^41^. The observed spectral similarity within Europe and the difference from the Asian samples, which must originate from difference in photonic nanoarchitectures, may be an indication of subtle divergence that can only be traced by fast-evolving DNA regions. The slightly different photonic nanoarchitectures being built in the scale cells of European an Asian *P. icarus* males are tentatively attributable to this divergence. As a first step in the investigation of this hypothesis, we investigated microsatellites—also called Single Sequence Repeats (SSRs)—in correlation with the optical characteristics of the dorsal wing scales of European male *P. icarus* butterflies. As SSRs are known to have exceptionally high mutation rate, these are good choice for tracing genetic variability of natural populations. A next level of analyses could be the application of genomic approaches that may provide new insights into the genetic architectures underlying color traits^42^.

Microsatellites are a popular and versatile marker type for ecological applications^43^. They have emerged as a very widely used choice for these studies in part because they have the potential to provide contemporary evaluation of migration and can estimate the relatedness of individuals. Many microsatellites have high-mutation rates (between 10^–6^ and 10^–2^ mutations per locus per generation), that generate the high levels of allelic diversity necessary for genetic studies of processes acting on ecological time scales^44^.

In the present paper, 80 *Polyommatus icarus* specimens, from four different locations (20 per each site) were used to correlate the spectral properties of the coloration of males within local populations with the genetic characteristics as revealed by DNA microsatellites. Our specific question is if we can find concordance between wing coloration and the fast-evolving SSR variability on a large geographic range.

## Materials and methods

### Sampling

The species *Polyommatus icarus* (Rottemburg, 1775) (English name: Common Blue) belongs to the family Gossamer-winged butterflies (Lycaenidae), and it is one of the most widespread Blue butterflies (Polyommatini) of the Eurasian landmass and is distributed from the Pacific to Atlantic coasts. It is not subjected to any restrictions: it is not threatened or endangered and collecting samples of this species is not disallowed. The specimens used in the present work (see Suppl. Table S1) were captured from wild populations in Taizé (TZ, Burgundy, Southern France, August 2017), two very close locations in the region of Érd (EF and EP, Central Hungary, May & August 2018) and Barațcoș (EB, Eastern Carpathians, Romania, July 2017). The distance between the westernmost and the easternmost sampling sites is of the order of 1600 km (see Suppl. Fig. S1). As the samples were intended for DNA extraction and spectral studies, they were not subjected to any chemical treatment or relaxed for setting in the standard preserving manner in collections. The four wings were removed from the body for precise spectral measurements, while the abdomens were used for the DNA extraction. The samples are stored in 20 × 20 × 5 mm plastic boxes individually and databased in the Institute of Technical Physics and Materials Science, Centre for Energy Research, Budapest, Hungary.

### Spectral measurements

Spectral measurements were carried out on the wings removed from the body, to allow precise measurements with an integrating sphere. This is necessary because the wings, due to the micro- and nanostructure of the individual scales, and to the 10–15 degrees deviation in the scale position from the wing plane, does not reflect light like a smooth, mirror-like surface. As a consequence, the measurements with the integrating sphere are more reliable than the data obtained in normal incidence^22^. All measurements were carried out with a modular fiber optic Avantes AvaSpec-HERO spectrophotometer (Avantes BV, Apeldoorn, Netherlands). The wing samples (all the four wings for each specimen) were illuminated by an Avantes DH-S-BAL (Avantes BV, Apeldoorn, Netherlands) UV–Vis light source (deuterium–halogen) through the illumination port of an integrating sphere (Avantes AvaSphere-30). The light reflected by the wings under any angle was collected by the integrating sphere and transmitted to the spectrometer. All the measurements were carried out with respect to an Avantes diffuse tile (Avantes WS-2) as a Lambertian, white standard used to set the 100% reflectance. Evaluation of the spectral data was carried out using Origin 2018 (OriginLab, Radnor, PA, USA) software.

### DNA extraction

DNA was extracted following the procedure described in Bereczki et al. (2014)^45^ by homogenizing the abdomen in 700 μl extraction buffer described by Gilbert et al. (2007)^46^ with 30 μl Proteinase K (20 mg/ml). The samples were incubated for 24 h at 55 °C followed by centrifugation at 14 000 rpm for 1 min. The DNA was precipitated by adding the mixture of 450 μl ammonium-acetate and storing the samples at − 20 °C for 30 min. The supernatant was washed twice with 600 μl of chloroform–isoamyl alcohol (24:1) to remove proteins. After centrifugation at 14,000 rpm for 5 min, 800 μl ice-cold ethanol was added to the supernatant and was stored at − 20 °C overnight. The precipitated DNA was pelletized by centrifugation at 14,000 rpm for 10 min at 4 °C. Following centrifugation, the supernatant was discarded, and the DNA pellet was washed twice with 96% ice-cold ethanol and stored at − 20 °C overnight. The pellet was air-dried for 1 h at 37 °C and was re-dissolved in 50 μl elution buffer (10 mM Tris–HCl, pH 8.0 and 0.5 mM EDTA, pH 9.0).

### Microsatellite development

As microsatellites are usually species-specific with limited transferability between species, we first searched the literature and SSR databases (e.g., https://data.ccmb.res.in/msdb/) for potential microsatellite loci that can be used in our target species. As we could not find any described primers for amplifying SSR loci in *Polyommatus icarus*, we developed them de novo using a next-generation sequencing approach^47^. A high-quality DNA extract was provided to a commercially available service of GenoScreen Inc. (Lille, France), who sequenced a standard Nextera Library on MiSeq machine (Illumina Inc., San Diego, CA, USA) for 300 bp long, paired-end reads. Paired reads were merged by USearch v.11.0.667^48^, and QDD v.3.1.2^49^ was used to mine potential SSR loci in silico from the Illumina reads (detailed screening criteria are described in Malkócs et al.^50^). Altogether, 50 potential microsatellite primer-pairs were tested in vivo for specific amplification in a polymerase chain reaction (PCR) using *Polyommatus icarus* DNA as template. The details of the PCR and selection of primers followed a general procedure of the Debrecen lab detailed elsewhere^50^, which resulted in selection of 10 primer pairs amplifying species-specific, apparently polymorphic microsatellite loci in our target species (Suppl. Table S2).

### Generation of microsatellite data

Twenty-two species-specific microsatellite loci (Suppl. Table S2) were amplified in PCR which contained the following reaction mixture: 2 × DreamTaq Green Buffer, 0.2 mM dNTP (each), 1 mg/ml bovine serum albumin, 0.5 μM of each primer, and 0.05 units DreamTaq Green DNA Polymerase in a final volume of 10 μl (all PCR reagents were purchased from Thermo Fisher Scientific, Carlsbad, California, USA). Cycling regime, programmed into an ABi Veriti PCR-machine (Applied Biosystems, Foster City, CA, USA), was denaturation at 95 °C for 2 min; 40 cycles of 15 s at 95 °C, 30 s at 62 °C, and 1 min at 72 °C; with a final extension step at 72 °C for 10 min. The forward primers were dyed using a fluorescent label (purchased from Applied Biosystems), and the success of the amplification was checked on an agarose-gel. Successful amplicons were capillary electrophoretized on an ABi 3130 Genetic Analyzer (Applied Biosystems). The products were multiplexed before loading them onto the genetic analyzer according to their predicted fragment length and fluorescent label type (Suppl. Table S3). The actual amount of PCR product that was added to the multiplex was assessed from band intensity (1.5–4 μl). This mixture was loaded to the capillary sequencer and raw electropherograms were analyzed by PeakScanner v.1.0 (Applied Biosystems).

### Analyses of microsatellite data

Genotypic data were imported into MS Excel (Microsoft, Redmond, WA, USA) and analyzed using GenAlEx v.6.5^51^. This software was used to assess deviation from the Hardy–Weinberg-equilibrium (HWe), to calculate population-level genetic diversity values and genetic differentiation (G_ST_). We tested for the presence of null-alleles in the software Micro-Checker v.2.2.3^52^. Individual level allelic frequency was used to visualize genetic distance between the samples genotyped in a Principal Component Analysis (PCA) as calculated in adegenet 2.1.2^53^. The same software was used to test an isolation-by-distance (IBD) pattern in our dataset in a Mantel-test where individual genetic distances based on the Bruvo-distance were tested in 10 k permutations against geographic distance measured in a Euclidean way. Finally, we run a Bayesian analysis of population structure as implemented in Structure v.2.3.4.^54^. We run the analysis for K = 1 to 4 groups, each grouping was tested in five independent runs, with 100,000 MCMC iterations as “burn-in” followed by 500,000 MCMC steps. The best K value (i.e., the number of genetic clusters present in the dataset) was assessed by the method of Evanno^55^ as implemented in the on-line version of Clumpak^56^. This software was also used in averaging across the five runs at each K.

## Results

### Spectral data

**Figure 2:**
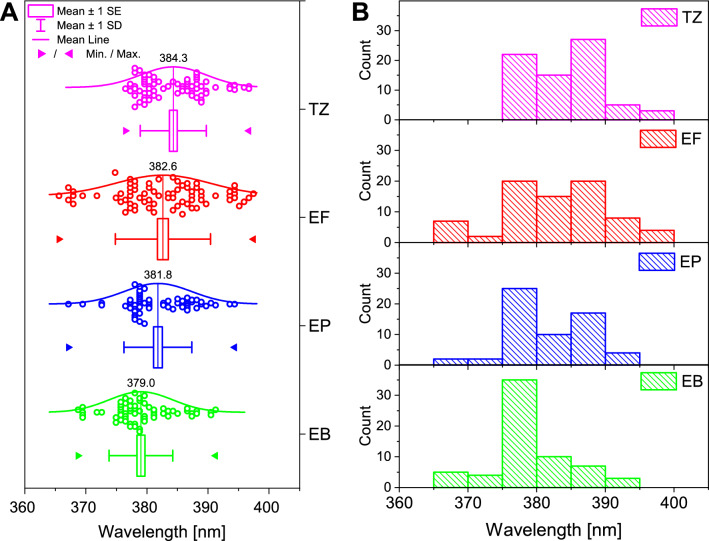
Cumulative presentation of the spectral position of the blue reflectance maximum for all four wings of male *Polyommatus icarus* butterflies. (**A**) The four sampling sites: TZ: Taizé (Burgundy, Southern France, August 2017); EF: Érd-1 (Central Hungary, May and August 2018); EP: Érd-2 (Central Hungary, May and August 2018); EB: Baraţcoş (Eastern Carpathians, Romania, July 2017). (**B**) Histograms of the spectral position of the blue reflectance maximum of the same specimens.

The reflectance data for the four dorsal wing surfaces of all male *Polyommatus icarus* (N_TZ_ = 18, N_EF_ = 19 N_EP_ = 15, N_EB_ = 16) measured in the present study were used to generate the statistical presentation in Fig. 2A. The individual measurement points represented as histograms are shown in Fig. 2B. One may observe from both kinds of representation that the color of the butterflies originating from the four sampling sites—the two extremes (TZ, elevation: 258 m and EB, elevation: 886 m) being at a distance of 1621 km, and the two nearest ones in Érd, Hungary at a distance of 700 m (EP and EF, elevation: 128 m) does show only a minor deviation from each other. In the case of the EB population situated at a higher altitude, the overwintering generation was sampled, whereas in the three other locations the summer generations (second and third were sampled). But even with this minor difference the median values of the measured spectral maxima fall in an interval of 5.3 nm.

### Microsatellite data

Altogether, we successfully genotyped 79 individuals of *Polyommatus icarus* for ten, de novo developed species-specific microsatellite loci from four populations representing three geographically distinct groups (i.e., Southern France, Central Hungary, Central Romania). These loci do not show significant presence of null-alleles at the genotyped loci, and none of them showed significant deviation from the HWe. Therefore, we could use them to characterize the neutral genetic variance of our studied populations. Allelic richness, genetic diversity and levels of inbreeding were found to be remarkably high in all studied populations with signs of homozygote access (Table 1).

**Table 1:** Allelic richness, genetic diversity, and levels of inbreeding in the studied *Polyommatus icarus* populations as reported by GenAlEx (mean ± s.e.).

Population	Na	Ho	He	uHe	Fis
EB (Barațcoș)	7.400 ± 1.204	0.627 ± 0.105	0.639 ± 0.100	0.655 ± 0.102	0.041 ± 0.036
EF (Érd)	7.400 ± 0.859	0.566 ± 0.089	0.623 ± 0.088	0.640 ± 0.091	0.085 ± 0.050
TZ (Taizé)	8.100 ± 1.441	0.550 ± 0.072	0.639 ± 0.082	0.656 ± 0.085	0.115 ± 0.053
EP (Érd)	7.700 ± 1.136	0.553 ± 0.076	0.631 ± 0.084	0.648 ± 0.086	0.120 ± 0.050

*Na* number of different alleles, *Ho* observed heterozygosity, *He* expected heterozygosity, *uHe* genetic diversity, *Fis* fixation index.

**Figure 3:**
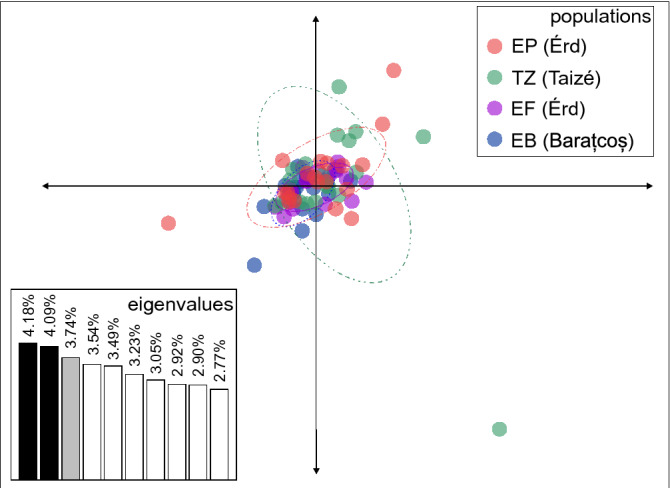
Principal component analysis of raw allelic frequencies of the studied *Polyommatus icarus* samples (populations are marked with different colors). 95% ellipses are superimposed on each population. Eigenvalues of the first ten axes are given in an inset barplot.

The ordination of genetic distance between individuals, calculated from allelic frequencies using PCA, resulted in representation of *ca.* 12% variance on the first three axes of which we show the ordination along the first two axes (Fig. 3). The individuals greatly overlap on the scatter plot, and, in spite of some satellite individuals that separate from their population, 95% confidence ellipses overlap in the center of the ordination.

Population genetic differentiation (G_ST_) was found to be exceptionally low between the four populations analyzed (Table 2). The only slightly significant difference (p = 0.029) was observed between the two populations located at the extreme western and eastern part of the sampling range (i.e., Taizé in France and Baraţcoş in Romania). In connection to the low level of genetic divergence we observed only slightly significant IBD pattern (r^2^ = 0.103, p = 0.016) at the individual level.

**Table 2:** Pairwise genetic differentiation values (G_ST_) below the diagonal, and their probability value based on 999 permutations above the diagonal as calculated in GenAlEx.

	EB	EF	TZ	EP
EB (Barațcoș)	–	0.163	0.029	0.486
EF (Érd)	0.003	–	0.120	0.609
TZ (Taizé)	0.007	0.004	–	0.493
EP (Érd)	0.000	0.001	0.000	–

**Figure 4:**
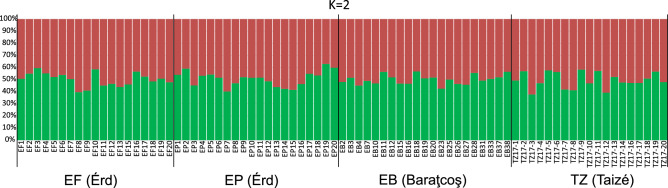
A posteriori assignment of the studied *Polyommatus icarus* individuals into two genetic clusters that is the most likely number of clusters in our genetic dataset as indicated by a structure analysis and its summary statistics generated by Clumpak.

Bayesian structuring of population genetic variability indicated the presence of two genetic clusters in our dataset (as indicated by the deltaK value). The a posteriori assignment of our individuals to these two clusters has, however, failed to find any structure in the dataset as all individuals were classified as belonging to either the first of the second cluster (Fig. 4). Their overall mean assignment approximated 50% in all four populations.

## Discussion

Our results show, in good agreement with earlier works^35^, that using neutral genetic markers it was not possible to find local differences between *Polyommatus icarus* populations from the western part of their main range of distribution (i.e., Western and Central-Eastern Europe). In concordance with this finding, the blue structural color of the males, used for sexual signaling, was similar in all four sampled sites in Europe and markedly different from the structural color of the males from the eastern part of the distribution (i.e., representing the eastern Palearctic).

Genetic data are in agreement with the measurement of physical color; we found almost no phylogeographic structure on the studied range of *Polyommatus icarus*. This is indicated by the overlap of 95% population means in genetic space (Fig. 3), the negligible level of genetic differentiation (Table 2), the marginally significant IBD pattern, and the lack of genetic structure within the genetic data (Fig. 4). As all loci were in HWe, we can safely conclude that what we detected was the neutral genetic variability of the populations studied, and this variability mirrors the pattern observed here in physical coloration. It is notable that we found remarkable genetic diversity on the ten microsatellite loci analyzed (Table 1), which may reflect the healthy nature of these populations. Although we observed a slight increase in homozygotes (Fis > 0) compared to the equal proportion of homozygotes and heterozygotes (Fis = 0), this may reflect a slight level of inbreeding. Such a genetic pattern can in fact be expected on the current range of distribution (i.e., within the territory of the Palearctic lineage as identified in^37,38^) in case of an extremely common butterfly species.

Despite the existence of different lineages as indicated by the available mitochondrial DNA data^37,38^, the structural blue coloration of male *Polyommatus icarus* specimens and their DNA microsatellites indicate that they constitute a single, large meta-population. This is in agreement with an earlier work, in which the allozymes of *P. icarus* were studied over a large part of Europe. The species had a rather high genetic diversity within populations, whereas differentiation between populations was very low^35^. Only a marginal trend of decline in genetic diversity from the south to the north was observed. We report here a similar pattern using microsatellites, although we could not find any cline in genetic diversity, probably due to low number of populations studied.

The value of the maximal spectral difference between the mean values of the four sampled sites of 5.3 nm, is about half of the mean standard deviation (SD) value for any of the sampled sites (Fig. 2A). This is in good agreement with the findings of our earlier work investigating the biogeographical patterns in the structural blue of male *Polyommatus icarus* butterflies^34^. In this work, we used museum samples. Because it was not allowed to use destructive sampling method for more than 300 specimens curated in museum collections, we used a setup specially designed for performing normal incidence, reproducible measurements on museum samples, the “spectroboard”^57^.

**Figure 5:**
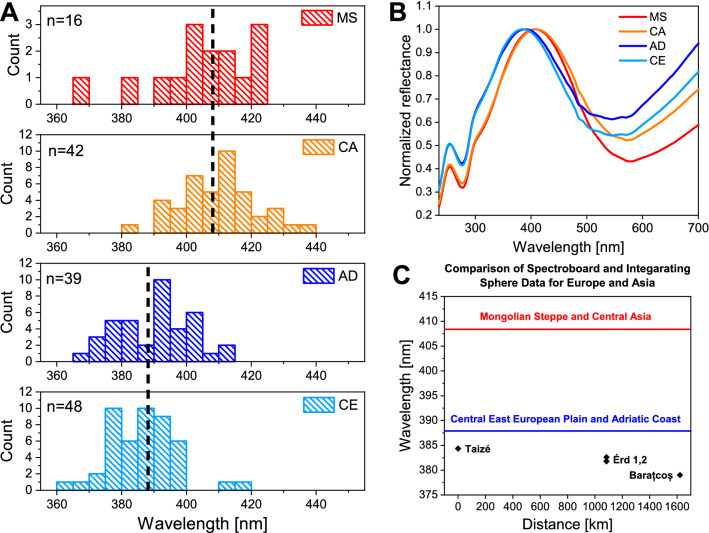
Comparison of spectroboard and integrating sphere measurements for Europe and Asia. (**A**) The histograms of the spectral position of reflectance maxima measured with spectroboard (wings not detached) are shown for four test regions: Mongolian Steppe (MS); Central Asia (CA); Adriatic Coast (AD); Central East European Plain (CE) from Ref.^34^. Number of specimens included in each region is indicated in each histogram in the right upper corner. (**B**) The spectra measured with spectroboard in normal incidence on museum samples averaged by the four test regions from Ref.^34^. In panel (**C**), the red line stands for the average of MS and CA regions, the blue line for the average of AD and CE regions (marked by broken lines in the left panel), the black diamonds show the spectral position of averaged reflectance maxima measured with the integrating sphere on the detached wings of the butterflies from the four samples regions: TZ, EF, EP, and EB.

To facilitate the comparison of earlier results with those of the present work, in Fig. 5 the average of the spectral positions of the four sampled sites and the average of the spectral data for the Central East European Plain and Adriatic Coast (CE and AD) and the Mongolian Steppe and Central Asia (MS and CA) from Ref.^34^ are compared. The two data sets clearly show that the normal incidence measurements taken with the spectroboard on set museum exemplars (as butterflies are usually presented in collections), without detaching the wings, and the measurements taken with the integrating sphere on detached wings of freshly captured butterflies, are coincident within a few nanometers. The comparison of the data in Fig. 5 and the data of Fig. 2 also shows that European *Polyommatus icarus* males have different coloration from the Asian ones.

As discussed earlier^58^, even minor alterations—of the order of 10 nm (see Suppl. Table 1S in^58^)—in the characteristic dimensions of the photonic nanoarchitecture responsible for the blue color of male *Polyommatus icarus* butterflies cause a shift in the spectral position of the reflectance maximum. This can be seen by the naked eye and can be well measured on the whole wing, or on single scales by microspectrometric methods. The structural differences induced a shift in the position of the blue reflectance maximum of 28 nm. This value exceeds by five times the difference found between the four sampling sites. In other words, the only 5 nm maximal difference between the spectral position of the blue maximum of the four sampled sites in Europe and the very good overlap of the range of the histograms in Fig. 2B indicates that the structure of the photonic nanoarchitecture responsible for the blue coloration is kept with a very high accuracy. Here, it has to be pointed out that the color generation mechanism of pigments and of photonic nanoarchitectures is different. While pigments produce color by the selective absorption of light, which is essentially dependent on the molecular structure of the pigment, photonic nanoarchitectures generate color by selective reflection, which is dependent on the refractive index contrast of the two transparent materials building up the nanocomposite (mainly chitin and air for butterfly wings) and the nanoscale dimensions of the structural elements of the nanoarchitecture^9^. The alteration of the second set of parameters—of the geometric dimensions—makes possible the variety of colors being generated by essentially very similar nanostructures^14^. Recently, we showed for the blue dorsal coloration of the *Polyommatus bellargus* males that this alteration of the nanoscale geometry may cause a shift in the spectral position of the blue reflectance maximum of 30 nm^59^. The color change is induced by the thickening of the chitin rich layers in the scale nanoarchitecture from 67.5 nm to 97.5 nm, while the thickness of the air rich layers was almost unchanged. Moreover, recently we shoved the blue color generating nanoarchitecture of *P. icarus* males can be tuned over a spectral range of 160 nm. We demonstrated experimentally and by model calculations, that by the conformal thinning of the photonic nanoarchitecture (by oxygen plasma etching), it is possible to blue shift the spectral position of the reflectance maximum, and by thickening (by atomic layer deposition ALD), it is possible to red shift the reflectance maximum^60^. The findings above convincingly show that the coincidence within a few nanometers of the averaged spectral positions of the blue reflectance maxima (Fig. 2) indicates that the characteristic dimensions of the photonic nanoarchitectures also must be similar within a few nanometers.

On the other hand, the structural color of *Polyommatus icarus* males exhibits remarkable stability on a time scale of 100 years^34^ and even after extreme alteration of the developmental process, like suspension of the pupal development for a duration of 10 weeks (the normal pupal stage has a duration of 8 to 10 days)^58^. Moreover, a similar structural color can be induced by prolonged cooling of the freshly formed pupae, for the *P. icarus*, females, too^58,61^.

As already mentioned in the Introduction, the scale formation takes place during the pupal stage and each scale develops individually from scale progenitor cells^62^, the Lycaenidae scales in the lower layer, called ground scales, in general have a simpler structure constituted of the network of ridges, cross-ribs and trabeculae and they contain melanin, a broad band absorber in the UV–Vis range. Melanin is responsible for the brown coloration of these scales. The top layer—the cover scales—contains the photonic nanoarchitectures, or pigments, eventually both. They are responsible for the often complex visual appearance of the butterfly wings. The nanoscale processes shaping the photonic nanoarchitectures inside the cover scales are not yet fully elucidated, despite several decades of investigations^23–26,28,29^. The (dead) chitinous scales that are each the product of a single precursor cell, offer a biologic system where phenotypic diversity can be studied cell by cell, both within and between species. Those scales reveal complex ultrastructures in the sub-micrometer range that are often connected to a photonic function, including iridescent blues and greens, highly reflective whites, or light-trapping blacks^33^. The shaping of the body of the scale^23^, including the ridge structure^25,33^ is governed primarily by cytoskeletal proteins like actin and fascin. Polymerization-depolymerization and organizing of actin filaments into complex networks are under the control of many actin associated proteins^63^. The cellular extensions of insect epithelial cells: bristles, hairs and scales are regarded as homologous structures that differ in morphology and function. They contain actin bundles that dictate their cellular morphology^64^. While the organization, function, and identity of the major actin-bundling proteins in bristles and hairs are known, this information on scales is unknown. It was shown recently that although scale and bristle are thought to be homologous structures, actin bundles have a differential requirement in shaping mosquito scales compared to bristles^65^.

As the protein synthesis is governed by the DNA in the cell nucleus, the remarkable coincidence in the spectral (optical) properties of *Polyommatus icarus* males collected in the four sampling sites indicate that despite the differences found between the mitochondrial DNA of the different European lineages established on the basis of COI data, the nuclear DNA governing the synthesis of the cytoskeletal proteins is highly similar. This is in good concordance with the result of the microsatellite investigation of the nuclear DNA of the butterflies collected in the four sampling sites. Both sets of data show the absence of major differences between the blue sexual signaling color of the male *P. icarus* butterflies collected in the four sampling sites and their nuclear DNA. Nevertheless, we need to emphasize that what have been studied so far is the neutral genetic variance, and it may not necessarily reflect traits that are under strong selection. Differences in structural color could also be coded epigenetically, and thus, only genomic or—more appropriately—transcriptomic investigations could unambiguously confirm the lack of differences. Despite this, the absence of differences in the optical properties exceeding 5 nm in the spectral position of the blue reflectance maximum, are a strong argument for the absence of such differences.

In the butterfly family Lycaenidae the phenomenon of reinforcement, understood as the avoiding of unfit hybrids and costly interspecific mating by enhancing natural selection based on prezygotic isolation between divergent populations or species, plays an important role^15^. Differences of male dorsal wing color are seen as the result of this reinforcement. As we showed earlier in the investigation of the wing coloration of nine lycaenid species inhabiting the same habitat in the environs of Budapest, the coloration of the males, and the photonic nanoarchitectures which generates their color, are both different enough that a neural network type software can efficiently identify the species with a better accuracy than 90%^14^. *Polyommatus icarus* is one of the nine investigated species and the males of all the nine species possess the same type of photonic nanoarchitecture: the so called “pepper-pot” type nanoarchitecture^65^. This is composed of perforated chitin layers stacked each over the other and separated by layers containing mostly air and a few pillars keeping the distance between the chitin layers (see Fig. 1 of Ref.^14^). Subtle differences in the geometric properties of these nanocomposites are responsible for the species-specific coloration of the dorsal wing surface of the males. However, it has to be emphasized, that all nine nanoarchitectures have structures like imposed by very similar “molds”. These molds may be produced inside the scale producing cells due to species-specific interlinking proteins, which determine the actin mold in which chitin will be deposited. After the chitin deposition, this mold is decomposed, and the cell dies leaving behind the chitin scale. When this process is completed, and air penetrates into the voids of the photonic nanoarchitecture, the blue color can be observed in the pupae (see Fig. 1).

## Conclusions

The blue sexual signaling coloration of the *Polyommatus icarus* males and ten nuclear DNA microsatellites for four sampling sites along a distance of 1600 km across Europe showed that the populations form a single evolutionary unit, which is in agreement with earlier results^35^. The structural coloration of the males is similar in all four sampled sites and differs characteristically from the coloration of Asian males. The excellent concordance of the coloration from Southern France, through Hungary, to the Carpathian Mountains in Romania is attributed to similar photonic nanoarchitectures being self-assembled individually in each dorsal wing cover scale in all 68 male specimens investigated (four wings per specimen, a total of 272 samples). It is argued that the chitin building up these species-specific photonic nanoarchitectures is deposited in protein-based molds, self-assembled in a process governed by DNA of the scale producing cell. This part of DNA is most probably under strong selection, thus, direct studying of this variation requires transcriptomic approaches.

## Supplementary Information

Supplementary Information.
