# Supplementary material for: Concordance of the spectral properties of dorsal wing scales with the phylogeographic structure of European male Polyommatus icarus butterflies
Source: Sci Rep. 2021 Aug 13;11:16498. doi: 10.1038/s41598-021-95881-z (PMC8363635; doi:10.1038/s41598-021-95881-z)
Supplement: Supplementary file 1 — Supplementary Information. [file 41598_2021_95881_MOESM1_ESM.docx]

**Supplementary Information on “Concordance of the Spectral Properties of Dorsal Wing Scales with the Phylogeographic Structure of European Male *Polyommatus icarus* Butterflies”**

Gábor Piszter, Krisztián Kertész, Gábor Sramkó, Virág Krízsik, Zsolt Bálint & László Péter Biró


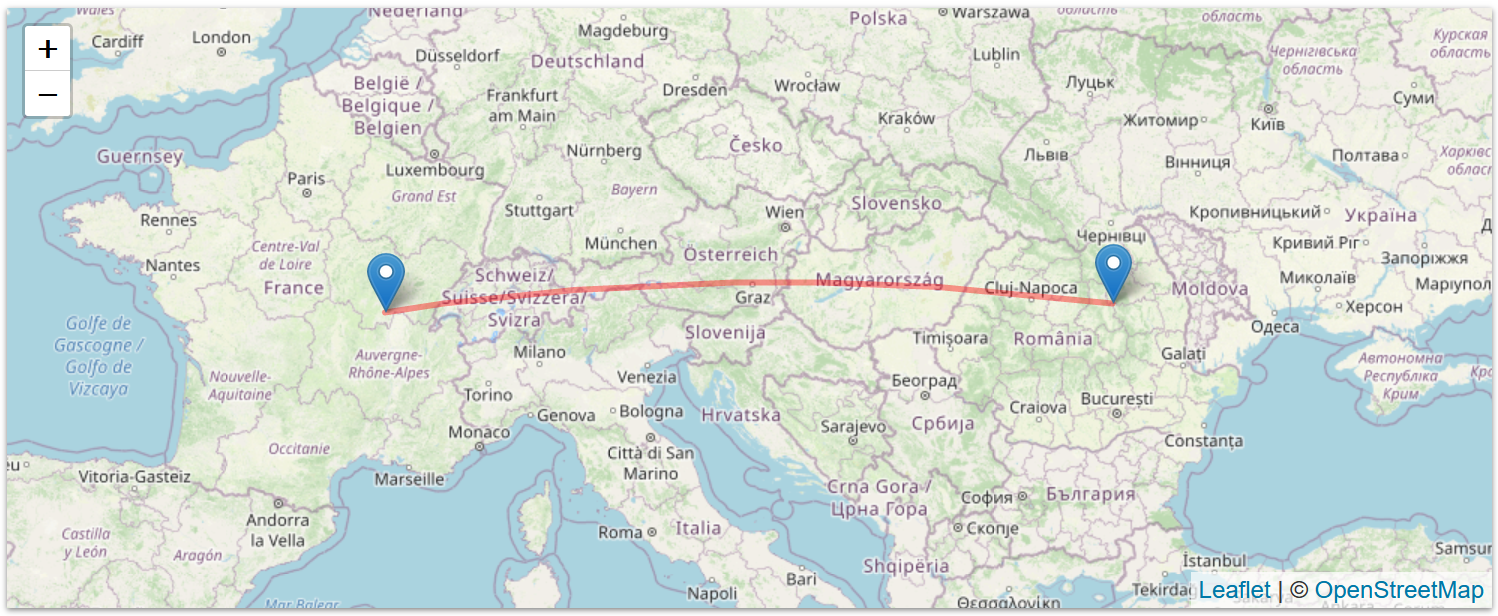


**Figure 1S. Geographic location of the sampled sites.** The left-hand blue marker indicates the location of Taizé (France), the two overlapping red dots indicate the two sampled sites in the environs of Érd (Hungary), the right-hand blue marker indicates the location of Barațcoș (Romania). The (flying) distance between Taizé and Barațcoș is 1621 km. Base map and data from OpenStreetMap and OpenStreetMap Foundation.

| **Sex** | **Geocoordinates** | **Country** | **Locality** | **Site (with acronym used in the main text)** | **Year** | **Month** | **Day** | **Collector(s)** |
| --- | --- | --- | --- | --- | --- | --- | --- | --- |
| male | 47°22'08.3"N 18°54'41.6"E | Hungary | Érd | Tollnok street (EF) | 2018 | 8 | 5 | Biró László Péter |
| male | 47°22'08.3"N 18°54'41.6"E | Hungary | Érd | Tollnok street (EF) | 2018 | 8 | 5 | Biró László Péter |
| male | 47°22'08.3"N 18°54'41.6"E | Hungary | Érd | Tollnok street (EF) | 2018 | 8 | 5 | Biró László Péter |
| male | 47°22'08.3"N 18°54'41.6"E | Hungary | Érd | Tollnok street (EF) | 2018 | 8 | 5 | Biró László Péter |
| male | 47°22'08.3"N 18°54'41.6"E | Hungary | Érd | Tollnok street (EF) | 2018 | 7 | 21 | Biró László Péter |
| male | 47°22'08.3"N 18°54'41.6"E | Hungary | Érd | Tollnok street (EF) | 2018 | 8 | 5 | Biró László Péter |
| male | 47°22'08.3"N 18°54'41.6"E | Hungary | Érd | Tollnok street (EF) | 2018 | 8 | 5 | Biró László Péter |
| male | 47°22'08.3"N 18°54'41.6"E | Hungary | Érd | Tollnok street (EF) | 2018 | 6 | 2 | Biró László Péter |
| male | 47°22'08.3"N 18°54'41.6"E | Hungary | Érd | Tollnok street (EF) | 2018 | 6 | 2 | Biró László Péter |
| male | 47°22'08.3"N 18°54'41.6"E | Hungary | Érd | Tollnok street (EF) | 2018 | 8 | 5 | Biró László Péter |
| male | 47°22'08.3"N 18°54'41.6"E | Hungary | Érd | Tollnok street (EF) | 2018 | 5 | 19 | Biró László Péter |
| male | 47°22'08.3"N 18°54'41.6"E | Hungary | Érd | Tollnok street (EF) | 2018 | 8 | 5 | Biró László Péter |
| male | 47°22'08.3"N 18°54'41.6"E | Hungary | Érd | Tollnok street (EF) | 2018 | 7 | 21 | Biró László Péter |
| male | 47°22'08.3"N 18°54'41.6"E | Hungary | Érd | Tollnok street (EF) | 2018 | 7 | 21 | Biró László Péter |
| male | 47°22'08.3"N 18°54'41.6"E | Hungary | Érd | Tollnok street (EF) | 2018 | 6 | 6 | Biró László Péter |
| male | 47°22'08.3"N 18°54'41.6"E | Hungary | Érd | Tollnok street (EF) | 2018 | 7 | 21 | Biró László Péter |
| male | 47°22'08.3"N 18°54'41.6"E | Hungary | Érd | Tollnok street (EF) | 2018 | 8 | 5 | Biró László Péter |
| male | 47°22'08.3"N 18°54'41.6"E | Hungary | Érd | Tollnok street (EF) | 2018 | 8 | 5 | Biró László Péter |
| female | 47°22'08.3"N 18°54'41.6"E | Hungary | Érd | Tollnok street (EF) | 2018 | 10 | 12 | Biró László Péter |
| male | 47°22'08.3"N 18°54'41.6"E | Hungary | Érd | Tollnok street (EF) | 2018 | 8 | 5 | Biró László Péter |
| male | 46°38'22.5"N 25°59'01.2"E | Romania | Lunca de Jos-Valea Rece | Barațcoș (EB) | 2017 | 6 | 14-16 | Bálint Zsolt & Biró László Péter |
| male | 46°38'22.5"N 25°59'01.2"E | Romania | Lunca de Jos-Valea Rece | Barațcoș (EB) | 2017 | 6 | 14-16 | Bálint Zsolt & Biró László Péter |
| male | 46°38'22.5"N 25°59'01.2"E | Romania | Lunca de Jos-Valea Rece | Barațcoș (EB) | 2017 | 6 | 14-16 | Bálint Zsolt & Biró László Péter |
| male | 46°38'22.5"N 25°59'01.2"E | Romania | Lunca de Jos-Valea Rece | Barațcoș (EB) | 2017 | 6 | 14-16 | Bálint Zsolt & Biró László Péter |
| male | 46°38'22.5"N 25°59'01.2"E | Romania | Lunca de Jos-Valea Rece | Barațcoș (EB) | 2017 | 6 | 14-16 | Bálint Zsolt & Biró László Péter |
| male | 46°38'22.5"N 25°59'01.2"E | Romania | Lunca de Jos-Valea Rece | Barațcoș (EB) | 2017 | 6 | 14-16 | Bálint Zsolt & Biró László Péter |
| male | 46°38'22.5"N 25°59'01.2"E | Romania | Lunca de Jos-Valea Rece | Barațcoș (EB) | 2017 | 6 | 14-16 | Bálint Zsolt & Biró László Péter |
| male | 46°38'22.5"N 25°59'01.2"E | Romania | Lunca de Jos-Valea Rece | Barațcoș (EB) | 2017 | 6 | 14-16 | Bálint Zsolt & Biró László Péter |
| male | 46°38'22.5"N 25°59'01.2"E | Romania | Lunca de Jos-Valea Rece | Barațcoș (EB) | 2017 | 6 | 14-16 | Bálint Zsolt & Biró László Péter |
| male | 46°38'22.5"N 25°59'01.2"E | Romania | Lunca de Jos-Valea Rece | Barațcoș (EB) | 2017 | 6 | 14-16 | Bálint Zsolt & Biró László Péter |
| male | 46°38'22.5"N 25°59'01.2"E | Romania | Lunca de Jos-Valea Rece | Barațcoș (EB) | 2017 | 6 | 14-16 | Bálint Zsolt & Biró László Péter |
| male | 46°38'22.5"N 25°59'01.2"E | Romania | Lunca de Jos-Valea Rece | Barațcoș (EB) | 2017 | 6 | 14-16 | Bálint Zsolt & Biró László Péter |
| male | 46°38'22.5"N 25°59'01.2"E | Romania | Lunca de Jos-Valea Rece | Barațcoș (EB) | 2017 | 6 | 14-16 | Bálint Zsolt & Biró László Péter |
| male | 46°38'22.5"N 25°59'01.2"E | Romania | Lunca de Jos-Valea Rece | Barațcoș (EB) | 2017 | 6 | 14-16 | Bálint Zsolt & Biró László Péter |
| male | 46°38'22.5"N 25°59'01.2"E | Romania | Lunca de Jos-Valea Rece | Barațcoș (EB) | 2017 | 6 | 14-16 | Bálint Zsolt & Biró László Péter |
| male | 46°38'22.5"N 25°59'01.2"E | Romania | Lunca de Jos-Valea Rece | Barațcoș (EB) | 2017 | 6 | 14-16 | Bálint Zsolt & Biró László Péter |
| male | 46°38'22.5"N 25°59'01.2"E | Romania | Lunca de Jos-Valea Rece | Barațcoș (EB) | 2017 | 6 | 14-16 | Bálint Zsolt & Biró László Péter |
| female | 46°38'22.5"N 25°59'01.2"E | Romania | Lunca de Jos-Valea Rece | Barațcoș (EB) | 2017 | 6 | 14-16 | Bálint Zsolt & Biró László Péter |
| female | 46°38'22.5"N 25°59'01.2"E | Romania | Lunca de Jos-Valea Rece | Barațcoș (EB) | 2017 | 6 | 14-16 | Bálint Zsolt & Biró László Péter |
| female | 46°38'22.5"N 25°59'01.2"E | Romania | Lunca de Jos-Valea Rece | Barațcoș (EB) | 2017 | 6 | 14-16 | Bálint Zsolt & Biró László Péter |
| female | 46°38'22.5"N 25°59'01.2"E | Romania | Lunca de Jos-Valea Rece | Barațcoș (EB) | 2017 | 6 | 14-16 | Bálint Zsolt & Biró László Péter |
| female | 47°22'20.1"N 18°54'12.5"E | Hungary | Érd | Mérnök street (EP) | 2018 | 8 | 17 | Biró László Péter |
| male | 47°22'20.1"N 18°54'12.5"E | Hungary | Érd | Mérnök street (EP) | 2018 | 8 | 16 | Biró László Péter |
| female | 47°22'20.1"N 18°54'12.5"E | Hungary | Érd | Mérnök street (EP) | 2018 | 8 | 16 | Biró László Péter |
| female | 47°22'20.1"N 18°54'12.5"E | Hungary | Érd | Mérnök street (EP) | 2018 | 8 | 3 | Biró László Péter |
| male | 47°22'20.1"N 18°54'12.5"E | Hungary | Érd | Mérnök street (EP) | 2018 | 8 | 16 | Biró László Péter |
| female | 47°22'20.1"N 18°54'12.5"E | Hungary | Érd | Mérnök street (EP) | 2018 | 8 | 16 | Biró László Péter |
| male | 47°22'20.1"N 18°54'12.5"E | Hungary | Érd | Mérnök street (EP) | 2018 | 8 | 3 | Biró László Péter |
| male | 47°22'20.1"N 18°54'12.5"E | Hungary | Érd | Mérnök street (EP) | 2018 | 8 | 3 | Biró László Péter |
| male | 47°22'20.1"N 18°54'12.5"E | Hungary | Érd | Mérnök street (EP) | 2018 | 8 | 16 | Biró László Péter |
| male | 47°22'20.1"N 18°54'12.5"E | Hungary | Érd | Mérnök street (EP) | 2018 | 8 | 16 | Biró László Péter |
| male | 47°22'20.1"N 18°54'12.5"E | Hungary | Érd | Mérnök street (EP) | 2018 | 8 | 16 | Biró László Péter |
| male | 47°22'20.1"N 18°54'12.5"E | Hungary | Érd | Mérnök street (EP) | 2018 | 7 | 26 | Biró László Péter |
| male | 47°22'20.1"N 18°54'12.5"E | Hungary | Érd | Mérnök street (EP) | 2018 | 8 | 16 | Biró László Péter |
| male | 47°22'20.1"N 18°54'12.5"E | Hungary | Érd | Mérnök street (EP) | 2018 | 8 | 16 | Biró László Péter |
| male | 47°22'20.1"N 18°54'12.5"E | Hungary | Érd | Mérnök street (EP) | 2018 | 8 | 16 | Biró László Péter |
| male | 47°22'20.1"N 18°54'12.5"E | Hungary | Érd | Mérnök street (EP) | 2018 | 8 | 16 | Biró László Péter |
| female | 47°22'20.1"N 18°54'12.5"E | Hungary | Érd | Mérnök street (EP) | 2018 | 8 | 16 | Biró László Péter |
| male | 47°22'20.1"N 18°54'12.5"E | Hungary | Érd | Mérnök street (EP) | 2018 | 8 | 3 | Biró László Péter |
| male | 47°22'20.1"N 18°54'12.5"E | Hungary | Érd | Mérnök street (EP) | 2018 | 8 | 3 | Biró László Péter |
| male | 47°22'20.1"N 18°54'12.5"E | Hungary | Érd | Mérnök street (EP) | 2018 | 8 | 3 | Biró László Péter |
| male | 46°30'06.7"N 4°39'54.0"E | France | Taizé | Le Bourg (TZ) | 2017 | 8 | 17 | Bálint Zsolt |
| male | 46°30'06.7"N 4°39'54.0"E | France | Taizé | Le Bourg (TZ) | 2017 | 8 | 15 | Bálint Zsolt |
| male | 46°30'06.7"N 4°39'54.0"E | France | Taizé | Le Bourg (TZ) | 2017 | 8 | 17 | Bálint Zsolt |
| male | 46°30'06.7"N 4°39'54.0"E | France | Taizé | Le Bourg (TZ) | 2017 | 8 | 17 | Bálint Zsolt |
| male | 46°30'06.7"N 4°39'54.0"E | France | Taizé | Le Bourg (TZ) | 2017 | 8 | 15 | Bálint Zsolt |
| male | 46°30'06.7"N 4°39'54.0"E | France | Taizé | Le Bourg (TZ) | 2017 | 8 | 17 | Bálint Zsolt |
| male | 46°30'06.7"N 4°39'54.0"E | France | Taizé | Le Bourg (TZ) | 2017 | 8 | 15 | Bálint Zsolt |
| male | 46°30'06.7"N 4°39'54.0"E | France | Taizé | Le Bourg (TZ) | 2017 | 8 | 15 | Bálint Zsolt |
| male | 46°30'06.7"N 4°39'54.0"E | France | Taizé | Le Bourg (TZ) | 2017 | 8 | 15 | Bálint Zsolt |
| male | 46°30'06.7"N 4°39'54.0"E | France | Taizé | Le Bourg (TZ) | 2017 | 8 | 15 | Bálint Zsolt |
| male | 46°30'06.7"N 4°39'54.0"E | France | Taizé | Le Bourg (TZ) | 2017 | 8 | 15 | Bálint Zsolt |
| male | 46°30'06.7"N 4°39'54.0"E | France | Taizé | Le Bourg (TZ) | 2017 | 8 | 17 | Bálint Zsolt |
| male | 46°30'06.7"N 4°39'54.0"E | France | Taizé | Le Bourg (TZ) | 2017 | 8 | 17 | Bálint Zsolt |
| female | 46°30'06.7"N 4°39'54.0"E | France | Taizé | Le Bourg (TZ) | 2017 | 8 | 17 | Bálint Zsolt |
| male | 46°30'06.7"N 4°39'54.0"E | France | Taizé | Le Bourg (TZ) | 2017 | 8 | 15 | Bálint Zsolt |
| female | 46°30'06.7"N 4°39'54.0"E | France | Taizé | Le Bourg (TZ) | 2017 | 8 | 15 | Bálint Zsolt |
| male | 46°30'06.7"N 4°39'54.0"E | France | Taizé | Le Bourg (TZ) | 2017 | 8 | 15 | Bálint Zsolt |
| male | 46°30'06.7"N 4°39'54.0"E | France | Taizé | Le Bourg (TZ) | 2017 | 8 | 17 | Bálint Zsolt |
| male | 46°30'06.7"N 4°39'54.0"E | France | Taizé | Le Bourg (TZ) | 2017 | 8 | 17 | Bálint Zsolt |
| male | 46°30'06.7"N 4°39'54.0"E | France | Taizé | Le Bourg (TZ) | 2017 | 8 | 15 | Bálint Zsolt |

**Table 1S. The list of specimens used in the present work.** Sex, collection location and date of the specimens are shown.

| **Locus** | **Primer sequences (5′–3′)** | **Repeat motif** | **Allele size range (bp)** | **Fluorescent dye** |
| --- | --- | --- | --- | --- |
| PICA1 | F: GATGACCTCTCGTCGTGTCC | (TG)9 | 266–292 | VIC |
|  | R: AGATCACACTCGATTATGTCAGCT |  |  |  |
| PICA2 | F: TGGACAATCGAGTGATGTATTAGCT | (CA)9 | 155–175 | 6-FAM |
|  | R: ACACGTACTTAGAGCTGGTATCG |  |  |  |
| PICA4 | F: CACCCTGTGATAAGATGTTACTGT | (TG)14 | 155* | VIC |
|  | R: AAATATGTGCAGGCGTCGC |  |  |  |
| PICA6 | F: GTCACATCCCTAGACGAGTGT | (TAAC)8 | 183* | NED |
|  | R: CGGAGGCCAAGATCCACTTT |  |  |  |
| PICA10 | F: TTCAGAGGAGAGTTTCGCGC | (AG)9 | 260–306 | NED |
|  | R: ATGTCTCATCCTTATCTAATACGTTCT |  |  |  |
| PICA11 | F: TCATCTCGCACTTGACTACGT | (TG)7 | 125* | PET |
|  | R: AAACTGAGGTAGACGTGCCT |  |  |  |
| PICA12 | F: TGCCCTAGATATCTACACAAATCGG | (ATT)7 | 132–168 | VIC |
|  | R: TTTGTAAGACGTGTTAGGTGGT |  |  |  |
| PICA16 | F: TTTCATGACATCTGGCGGGT | (GT)7 | 84–116 | 6-FAM |
|  | R: TTCTACCTGCTCGACGGTTG |  |  |  |
| PICA19 | F: TCCATGGCAAATTTCAGCAAA | (ACAG)7 | 255* | PET |
|  | R: CCCTCTTCATTAATCTTTCTGCC |  |  |  |
| PICA21 | F: TCGGCCATTACTGTATGACGT | (ATC)7 | 181–196 | NED |
|  | R: CTCCTATACATGGTGAGTGGCG |  |  |  |
| PICA22 | F: TGGATGCTTGGACTTGGGAG | (TG)9 | 274* | VIC |
|  | R: GCAGTTCATTATAAATTGAAAGCTGT |  |  |  |
| PICA23 | F: TCTTTGTAACCCATGTTTCATTATTGA | (TTA)7 | 100–145 | 6-FAM |
|  | R: ACATACACATACGCACCCTACAA |  |  |  |
| PICA24 | F: CAGTTGTTTCCCAGGCACG | (CA)7 | 141–169 | NED |
|  | R: GGTAGTGGCATTCAACACCG |  |  |  |
| PICA25 | F: AGTGGTGCCAGAATAATGAGCA | (TCA)7 | 298* | 6-FAM |
|  | R: ACACTAGCCACATCTTTAGCAGT |  |  |  |
| PICA27 | F: ATGCGCGGATATTGTGTTCC | (GT)9 | 95–149 | 6-FAM |
|  | R: CACATTTCCTTCCCAAGCGG |  |  |  |
| PICA30 | F: TCTGATCGGAGCTACGTCCT | (TC)9 | 232* | 6-FAM |
|  | R: TGTGATCTGGTGATAAATGTATAGGT |  |  |  |
| PICA34 | F: GCATCTTCAATGTGAACTATCAAAC | (AC)7 | 289* | VIC |
|  | R: ACGTAAGGTGGGTAGAGGAAGA |  |  |  |
| PICA36 | F: CGGCCGTCTGATAATACGCT | (AC)10 | 159* | VIC |
|  | R: ACCTCTGTCCCAAGAGTCTT |  |  |  |
| PICA37 | F: GGCACCAGAGGTAGCACAAA | (CA)8 | 130* | PET |
|  | R: CCGGCCAGGGTCCATACAAA |  |  |  |
| PICA39 | F: TGCGACTATGAAAGCAACACA | (TTTC)8 | 189* | PET |
|  | R: TACGGGCGTCACTTGGTAGT |  |  |  |
| PICA42 | F: ATGCGAGTCCACGACTACTT | (TG)8 | 171–183 | 6-FAM |
|  | R: CAACACATTGAGCCAGTCGG |  |  |  |
| PICA45 | F: CCGATGGGCTGTTCTACCAG | (GGTGA)7 | 226* | PET |
|  | R: GAAAGCCTTCGCGCGAATTA |  |  |  |
| *Predicted size from in-silico primer design step. |  |  |  |  |

**Table 2S. Characteristics of 22 microsatellite loci designed in *Polyommatus icarus*.**

| **Sample** | **PICA16** |  | **PICA12** |  | **PICA2** |  | **PICA1** |  | **PICA23** |  | **PICA24** |  | **PICA10** |  | **PICA27** |  | **PICA42** |  | **PICA21** |  |
| --- | --- | --- | --- | --- | --- | --- | --- | --- | --- | --- | --- | --- | --- | --- | --- | --- | --- | --- | --- | --- |
| **EB2** | 96 | 100 | 141 | 141 | 155 | 167 | 266 | 278 | 109 | 115 | 167 | 167 | 288 | 294 | 109 | 113 | 177 | 179 | 181 | 190 |
| **EB3** | 94 | 94 | 141 | 141 | 157 | 169 | 272 | 282 | 115 | 115 | 167 | 167 | 286 | 290 | 0 | 0 | 173 | 179 | 190 | 190 |
| **EB4** | 98 | 112 | 141 | 141 | 161 | 161 | 266 | 272 | 115 | 118 | 167 | 167 | 290 | 296 | 111 | 133 | 179 | 181 | 184 | 184 |
| **EB7** | 100 | 102 | 144 | 150 | 157 | 159 | 270 | 280 | 103 | 118 | 167 | 167 | 290 | 292 | 113 | 113 | 177 | 177 | 190 | 190 |
| **EB10** | 96 | 106 | 141 | 141 | 165 | 165 | 268 | 274 | 103 | 115 | 167 | 167 | 282 | 288 | 117 | 127 | 175 | 175 | 190 | 190 |
| **EB11** | 92 | 94 | 141 | 141 | 157 | 165 | 266 | 278 | 118 | 118 | 167 | 167 | 290 | 296 | 115 | 119 | 177 | 179 | 190 | 190 |
| **EB12** | 96 | 104 | 141 | 150 | 155 | 159 | 270 | 274 | 115 | 115 | 167 | 167 | 282 | 288 | 111 | 135 | 179 | 181 | 190 | 190 |
| **EB15** | 96 | 102 | 141 | 141 | 161 | 163 | 272 | 278 | 106 | 118 | 167 | 167 | 284 | 292 | 0 | 0 | 179 | 179 | 190 | 190 |
| **EB16** | 94 | 100 | 141 | 150 | 155 | 161 | 268 | 282 | 109 | 118 | 167 | 167 | 288 | 292 | 121 | 139 | 179 | 181 | 190 | 190 |
| **EB18** | 96 | 98 | 141 | 141 | 159 | 163 | 270 | 274 | 109 | 115 | 167 | 167 | 288 | 288 | 119 | 129 | 177 | 179 | 190 | 190 |
| **EB19** | 94 | 96 | 141 | 141 | 155 | 161 | 274 | 280 | 115 | 118 | 167 | 167 | 290 | 302 | 117 | 125 | 179 | 179 | 190 | 190 |
| **EB20** | 92 | 98 | 144 | 150 | 159 | 165 | 266 | 268 | 103 | 118 | 167 | 167 | 288 | 288 | 117 | 117 | 179 | 179 | 190 | 196 |
| **EB23** | 100 | 112 | 141 | 141 | 155 | 161 | 272 | 280 | 112 | 136 | 167 | 167 | 292 | 294 | 109 | 117 | 181 | 181 | 190 | 190 |
| **EB25** | 98 | 102 | 141 | 168 | 159 | 161 | 270 | 274 | 109 | 118 | 167 | 167 | 294 | 294 | 115 | 117 | 179 | 181 | 190 | 190 |
| **EB26** | 96 | 100 | 141 | 150 | 159 | 159 | 270 | 278 | 112 | 112 | 167 | 167 | 286 | 292 | 109 | 131 | 175 | 179 | 184 | 190 |
| **EB27** | 94 | 98 | 141 | 150 | 157 | 161 | 266 | 272 | 112 | 112 | 167 | 167 | 286 | 296 | 123 | 125 | 171 | 179 | 190 | 190 |
| **EB28** | 96 | 100 | 141 | 141 | 159 | 159 | 274 | 280 | 115 | 118 | 167 | 167 | 288 | 290 | 111 | 121 | 173 | 181 | 190 | 190 |
| **EB31** | 88 | 96 | 141 | 141 | 159 | 165 | 270 | 280 | 106 | 109 | 167 | 167 | 286 | 290 | 113 | 119 | 175 | 177 | 190 | 190 |
| **EB33** | 94 | 98 | 141 | 141 | 157 | 159 | 280 | 288 | 106 | 118 | 167 | 167 | 290 | 290 | 111 | 111 | 179 | 181 | 190 | 193 |
| **EB37** | 96 | 100 | 141 | 141 | 159 | 161 | 268 | 280 | 109 | 115 | 167 | 167 | 284 | 284 | 119 | 121 | 177 | 179 | 190 | 190 |
| **EB38** | 92 | 94 | 141 | 141 | 159 | 159 | 270 | 270 | 112 | 118 | 167 | 167 | 284 | 288 | 111 | 113 | 173 | 179 | 190 | 190 |
| **EF1** | 98 | 102 | 141 | 150 | 159 | 169 | 274 | 284 | 118 | 118 | 167 | 167 | 282 | 288 | 117 | 117 | 179 | 179 | 190 | 190 |
| **EF2** | 84 | 98 | 141 | 141 | 159 | 163 | 270 | 276 | 112 | 112 | 167 | 167 | 288 | 288 | 115 | 115 | 179 | 179 | 190 | 190 |
| **EF3** | 96 | 98 | 141 | 141 | 155 | 157 | 270 | 278 | 103 | 115 | 167 | 167 | 288 | 288 | 111 | 119 | 179 | 179 | 190 | 190 |
| **EF4** | 98 | 106 | 141 | 141 | 165 | 165 | 272 | 278 | 115 | 118 | 167 | 167 | 290 | 296 | 117 | 117 | 179 | 179 | 190 | 190 |
| **EF5** | 96 | 100 | 141 | 141 | 163 | 165 | 266 | 282 | 109 | 118 | 167 | 167 | 288 | 290 | 105 | 119 | 179 | 179 | 190 | 190 |
| **EF6** | 96 | 96 | 141 | 141 | 159 | 161 | 278 | 280 | 112 | 115 | 167 | 167 | 0 | 0 | 109 | 113 | 173 | 177 | 190 | 190 |
| **EF7** | 92 | 92 | 150 | 153 | 159 | 159 | 274 | 292 | 115 | 115 | 167 | 167 | 288 | 288 | 115 | 117 | 179 | 181 | 184 | 190 |
| **EF8** | 86 | 96 | 153 | 156 | 159 | 161 | 276 | 278 | 106 | 112 | 167 | 167 | 288 | 296 | 111 | 113 | 175 | 179 | 190 | 190 |
| **EF9** | 94 | 98 | 132 | 141 | 159 | 167 | 268 | 276 | 100 | 100 | 167 | 167 | 288 | 290 | 117 | 133 | 179 | 181 | 190 | 193 |
| **EF10** | 98 | 100 | 150 | 150 | 0 | 0 | 268 | 272 | 115 | 115 | 167 | 167 | 288 | 290 | 113 | 115 | 179 | 179 | 190 | 193 |
| **EF11** | 96 | 96 | 141 | 144 | 159 | 161 | 266 | 272 | 109 | 121 | 167 | 167 | 282 | 290 | 109 | 131 | 179 | 179 | 193 | 193 |
| **EF12** | 98 | 98 | 141 | 141 | 161 | 161 | 268 | 276 | 100 | 103 | 167 | 167 | 288 | 292 | 127 | 127 | 177 | 179 | 190 | 190 |
| **EF13** | 100 | 106 | 144 | 144 | 161 | 163 | 268 | 280 | 109 | 109 | 167 | 167 | 290 | 292 | 109 | 119 | 179 | 181 | 190 | 190 |
| **EF15** | 96 | 98 | 141 | 141 | 161 | 169 | 272 | 274 | 112 | 118 | 167 | 169 | 288 | 288 | 133 | 133 | 177 | 179 | 190 | 196 |
| **EF16** | 98 | 100 | 141 | 141 | 159 | 161 | 266 | 284 | 109 | 109 | 167 | 167 | 288 | 290 | 111 | 119 | 179 | 179 | 190 | 190 |
| **EF17** | 100 | 100 | 141 | 141 | 157 | 161 | 272 | 274 | 112 | 121 | 167 | 167 | 286 | 290 | 117 | 127 | 179 | 179 | 190 | 190 |
| **EF18** | 98 | 102 | 141 | 147 | 159 | 165 | 272 | 280 | 109 | 115 | 167 | 167 | 278 | 282 | 111 | 117 | 179 | 179 | 190 | 190 |
| **EF19** | 86 | 96 | 141 | 150 | 157 | 159 | 272 | 280 | 115 | 115 | 167 | 167 | 288 | 288 | 111 | 127 | 179 | 183 | 190 | 190 |
| **EF20** | 92 | 92 | 141 | 141 | 161 | 167 | 268 | 272 | 112 | 115 | 167 | 167 | 290 | 290 | 113 | 121 | 179 | 183 | 187 | 190 |
| **TZ1** | 100 | 104 | 141 | 150 | 155 | 157 | 272 | 280 | 115 | 115 | 167 | 167 | 288 | 290 | 105 | 131 | 177 | 179 | 190 | 190 |
| **TZ2** | 92 | 98 | 141 | 141 | 155 | 157 | 272 | 278 | 115 | 115 | 167 | 167 | 288 | 288 | 113 | 135 | 179 | 179 | 190 | 193 |
| **TZ3** | 100 | 106 | 141 | 153 | 155 | 161 | 280 | 290 | 115 | 115 | 167 | 167 | 276 | 288 | 95 | 117 | 177 | 179 | 196 | 196 |
| **TZ4** | 92 | 96 | 141 | 141 | 157 | 159 | 0 | 0 | 112 | 115 | 167 | 167 | 260 | 298 | 121 | 121 | 177 | 181 | 190 | 190 |
| **TZ5** | 96 | 98 | 141 | 141 | 155 | 159 | 278 | 278 | 112 | 115 | 167 | 167 | 290 | 290 | 119 | 119 | 179 | 179 | 184 | 193 |
| **TZ6** | 98 | 100 | 141 | 141 | 159 | 165 | 266 | 266 | 115 | 118 | 167 | 167 | 286 | 294 | 109 | 115 | 177 | 179 | 190 | 190 |
| **TZ7** | 106 | 108 | 141 | 150 | 155 | 157 | 278 | 278 | 133 | 145 | 167 | 167 | 286 | 288 | 115 | 125 | 179 | 179 | 190 | 190 |
| **TZ8** | 104 | 104 | 141 | 162 | 159 | 167 | 266 | 278 | 103 | 106 | 167 | 167 | 288 | 288 | 127 | 129 | 179 | 179 | 190 | 190 |
| **TZ9** | 98 | 98 | 141 | 150 | 159 | 159 | 270 | 272 | 112 | 112 | 167 | 167 | 282 | 294 | 109 | 115 | 179 | 179 | 190 | 190 |
| **TZ10** | 96 | 96 | 141 | 150 | 155 | 163 | 278 | 286 | 115 | 115 | 165 | 167 | 282 | 288 | 119 | 133 | 179 | 179 | 190 | 190 |
| **TZ11** | 98 | 98 | 141 | 141 | 157 | 165 | 268 | 280 | 112 | 112 | 167 | 167 | 282 | 282 | 113 | 117 | 177 | 177 | 190 | 190 |
| **TZ12** | 88 | 88 | 141 | 144 | 159 | 159 | 278 | 278 | 100 | 103 | 165 | 167 | 282 | 288 | 121 | 139 | 179 | 179 | 190 | 190 |
| **TZ13** | 94 | 106 | 141 | 141 | 159 | 165 | 270 | 270 | 112 | 115 | 167 | 167 | 282 | 282 | 119 | 127 | 177 | 177 | 184 | 190 |
| **TZ14** | 104 | 104 | 141 | 141 | 157 | 157 | 266 | 270 | 103 | 103 | 167 | 167 | 288 | 288 | 119 | 119 | 173 | 179 | 187 | 190 |
| **TZ16** | 94 | 98 | 141 | 144 | 155 | 161 | 274 | 278 | 112 | 112 | 167 | 167 | 280 | 288 | 105 | 105 | 179 | 179 | 184 | 190 |
| **TZ17** | 96 | 98 | 141 | 141 | 159 | 163 | 266 | 280 | 115 | 142 | 167 | 167 | 282 | 288 | 127 | 145 | 177 | 177 | 190 | 190 |
| **TZ18** | 96 | 98 | 141 | 144 | 159 | 159 | 268 | 282 | 100 | 112 | 167 | 167 | 288 | 292 | 111 | 113 | 177 | 179 | 190 | 190 |
| **TZ19** | 94 | 98 | 141 | 141 | 161 | 165 | 272 | 274 | 109 | 109 | 167 | 167 | 286 | 288 | 115 | 117 | 173 | 179 | 190 | 193 |
| **TZ20** | 92 | 116 | 141 | 150 | 0 | 0 | 266 | 268 | 112 | 115 | 167 | 167 | 282 | 288 | 113 | 137 | 177 | 177 | 190 | 190 |
| **EP1** | 94 | 100 | 141 | 141 | 159 | 161 | 278 | 278 | 109 | 112 | 167 | 167 | 286 | 296 | 121 | 121 | 179 | 179 | 190 | 190 |
| **EP2** | 92 | 94 | 141 | 141 | 155 | 155 | 268 | 272 | 115 | 115 | 167 | 167 | 286 | 288 | 115 | 115 | 179 | 179 | 190 | 193 |
| **EP3** | 92 | 102 | 144 | 144 | 159 | 163 | 266 | 278 | 112 | 118 | 167 | 167 | 292 | 292 | 111 | 127 | 179 | 179 | 190 | 190 |
| **EP4** | 94 | 94 | 141 | 141 | 159 | 159 | 274 | 282 | 106 | 109 | 167 | 167 | 282 | 286 | 117 | 117 | 179 | 181 | 190 | 190 |
| **EP5** | 94 | 96 | 141 | 162 | 161 | 161 | 274 | 274 | 103 | 109 | 167 | 167 | 288 | 288 | 113 | 115 | 177 | 179 | 190 | 190 |
| **EP6** | 92 | 98 | 141 | 153 | 159 | 173 | 272 | 272 | 109 | 118 | 167 | 167 | 286 | 288 | 113 | 121 | 179 | 179 | 190 | 190 |
| **EP7** | 104 | 108 | 141 | 165 | 159 | 165 | 268 | 278 | 103 | 115 | 167 | 167 | 288 | 288 | 111 | 123 | 183 | 183 | 190 | 190 |
| **EP8** | 98 | 104 | 141 | 141 | 155 | 163 | 270 | 270 | 115 | 118 | 167 | 167 | 290 | 290 | 123 | 123 | 177 | 179 | 190 | 196 |
| **EP9** | 94 | 102 | 141 | 141 | 157 | 159 | 268 | 278 | 112 | 115 | 167 | 167 | 280 | 288 | 111 | 133 | 179 | 179 | 190 | 190 |
| **EP10** | 96 | 96 | 141 | 141 | 159 | 165 | 270 | 270 | 115 | 118 | 167 | 167 | 288 | 294 | 103 | 119 | 177 | 181 | 190 | 193 |
| **EP11** | 94 | 94 | 141 | 168 | 155 | 159 | 266 | 280 | 109 | 112 | 0 | 0 | 0 | 0 | 113 | 113 | 179 | 181 | 190 | 193 |
| **EP12** | 98 | 100 | 141 | 141 | 165 | 175 | 268 | 272 | 106 | 112 | 167 | 167 | 288 | 288 | 115 | 125 | 177 | 179 | 190 | 196 |
| **EP13** | 96 | 100 | 141 | 141 | 0 | 0 | 268 | 272 | 106 | 109 | 167 | 167 | 284 | 288 | 129 | 135 | 179 | 183 | 190 | 190 |
| **EP14** | 96 | 100 | 141 | 153 | 159 | 163 | 282 | 284 | 109 | 109 | 167 | 167 | 0 | 0 | 111 | 137 | 0 | 0 | 190 | 190 |
| **EP15** | 96 | 110 | 141 | 141 | 159 | 163 | 266 | 280 | 118 | 121 | 167 | 167 | 294 | 306 | 115 | 149 | 179 | 179 | 190 | 190 |
| **EP16** | 92 | 92 | 150 | 150 | 157 | 159 | 266 | 270 | 115 | 118 | 141 | 147 | 0 | 0 | 121 | 121 | 179 | 179 | 190 | 193 |
| **EP17** | 92 | 98 | 0 | 0 | 157 | 165 | 270 | 270 | 112 | 115 | 167 | 167 | 280 | 288 | 119 | 121 | 179 | 181 | 190 | 190 |
| **EP18** | 98 | 106 | 141 | 150 | 155 | 159 | 266 | 274 | 106 | 115 | 167 | 169 | 288 | 288 | 113 | 119 | 179 | 179 | 190 | 190 |
| **EP19** | 98 | 100 | 141 | 150 | 157 | 159 | 274 | 278 | 115 | 115 | 167 | 167 | 288 | 288 | 115 | 115 | 179 | 179 | 190 | 190 |
| **EP20** | 94 | 98 | 141 | 141 | 159 | 161 | 266 | 266 | 112 | 112 | 167 | 167 | 290 | 292 | 115 | 115 | 177 | 179 | 190 | 190 |

**Table 3S. Raw genotyping data of ten microsatellite loci used in this study.**
